# The Double-Edged Sword of Type 17 Immunity in Wound Healing and Skin Barrier Repair: Microenvironment-Driven Functional Plasticity

**DOI:** 10.3390/biom16030414

**Published:** 2026-03-11

**Authors:** Yao Lu, Fuxin Xu, Fazhi Qi, Yuyan Pan

**Affiliations:** 1 Department of Plastic Surgery, Fudan University Affiliated Zhongshan Hospital, Shanghai 200032, China; 17301050253@fudan.edu.cn; 2 Plastic Surgery Hospital, Chinese Academy of Medical Sciences and Peking Union Medical College, Beijing 100144, China; 3 Department of General Surgery, Fudan University Affiliated Huadong Hospital, Shanghai 200040, China; 24211280009@m.fudan.edu.cn

**Keywords:** IL-17 signaling, Th17 cell plasticity, wound healing, skin barrier repair, microenvironmental regulation

## Abstract

Type 17 immune responses are primarily mediated by Th17 cells and their effector cytokine interleukin-17 (IL-17), exerting a dual influence on wound healing. IL-17 plays a protective role during the initial stages of acute injury by facilitating rapid neutrophil recruitment, inducing antimicrobial peptide production and reinforcing pro-inflammatory signaling. However, sustained high signal of IL-17 results in a persistent inflammatory response that impairs keratinocyte proliferation and migration, angiogenesis, and nerve regeneration. This review elucidates the IL-17 signal effects and Th17 subset plasticity, which determines wound healing and skin barrier repair through their interactions with microbiota–immune, neuro–immune and metabolic reprogramming systems. Finally, we propose that the new therapeutic methods focus on IL-17 targets through precise spatiotemporal modulation and microenvironmental remodeling to create effective treatments for chronic non-healing wounds.

## 1. Introduction

Wound healing and barrier repair are complex biological processes. Wound healing proceeds through three dynamically overlapping phases: inflammation, proliferation, and remodeling [1]. The progression of this entire process requires exact timing and location control among multiple cell types, including platelets, immune cells and resident stromal cells [2,3]. An acute wound progresses through the orderly, time-dependent phases, while a chronic wound is often disrupted because of the local or systemic infections, ischemia or metabolic problems, and remains unhealed beyond 12 weeks [4]. Chronic wounds or non-healing wound—most notably diabetic foot ulcers (DFUs), which serve as the primary focus of the present study—are characterized by sustained inflammation, excessive activation of matrix metalloproteinases (MMPs), and defective re-epithelialization, because immune cells fail to transform properly into repair-promoting types [3,5]. Skin barrier repair extends beyond wound closure; it also includes the restoration of the physical barrier (e.g., the stratum corneum and intercellular tight junctions) and the functional immune barrier (e.g., immune regulation and microbiome equilibrium). Therefore, studies are focusing on controlling immune cell phenotypic transformation and related cytokines, as well as microenvironment homeostasis, which has become a critical objective in overcoming chronic non-healing wounds and barrier-associated dermatological disorders.

Research on wound healing demonstrates macrophages and their phenotypic polarization during tissue repair [6,7]. The initial inflammatory stage involves macrophages, which become classically activated (M1) to perform pathogen elimination and dead tissue removal through their production of IL-1β, IL-6 and TNF-α and their high iNOS expression [8]. The healing process needs macrophages to transform into M2 cells because this transition enables managing inflammation while helping tissue repair through blood vessel formation and matrix structure adjustment [9,10]. Recently evidences indicate that Type 17 immune responses serve as essential protective systems that maintain both skin and mucosal homeostasis [11,12]. The IL-17 cytokine family stands as the main focus of this answer, among which IL-17A and IL-17F represent the two most researched members of the A–F isoforms. The immune response of Type 17 cells in skin tissue shows a similar dual effect to macrophage functions because it protects against outside threats but can lead to prolonged inflammation, damaging tissues and disrupting skin barrier repair [13,14].

The research investigates the spatiotemporal dynamics of Type 17 immune responses, while concentrating on the Th17/IL-17 axis functions, which enable wound recovery and protect skin integrity. We explain how cellular origin, local cytokine levels, and IL-17 signaling duration determine its functional effects, leading to protective immunity or pathogenic inflammation. Meanwhile this review demonstrates that plasticity of Th17 cells depends on microbiota–immune interactions, neuro–immune crosstalk, and metabolic reprogramming. Finally, we assess the therapies of targeting the IL-17 axis and propose immunomodulatory strategies based on precise spatiotemporal intervention to improve clinical outcomes in patients with chronic wounds.

## 2. The IL-17 Family and Type 17 Immune Responses

### 2.1. Members of the IL-17 Family and Signaling Pathways

As the first member of the IL-17 family, IL-17A was found in 1993 [15] and initially identified in 1995 [16], commonly referred to as IL-17. The discovery of Th17 cells was published in 2005 [17,18] established a new understanding of helper T cell biology by overthrowing the established Th1/Th2 model, which had ruled for twenty years. The IL-17 cytokine family consists of six related ligands (IL-17A to IL-17F) and five receptor subunits (IL-17RA to IL-17RE) [19]. IL-17A is frequently co-expressed with IL-17F, sharing approximately 50% sequence homology [20]. These cytokines signal as homodimers or as IL-17A/F heterodimers whose structures share a cysteine-knot protein. IL-17C is mainly produced from epithelial cells, as an autocrine epithelial stabilizer acting through IL-17RA/RE to reinforce barrier immunity [21] (Table 1). The family also includes IL-25, a Type 2 response inducer acting through IL-17RA/IL-17RB, and other members like IL-17B and IL-17D, not primarily associated with classical Type 17 immune responses in cutaneous injury, which are not discussed in detail here due to limited understanding of their signaling mechanisms and physiological roles. This review focuses on the IL-17A/F because of their potent pro-inflammatory effects and well-characterized roles in chronic wound microenvironments.

**Figure 1:**
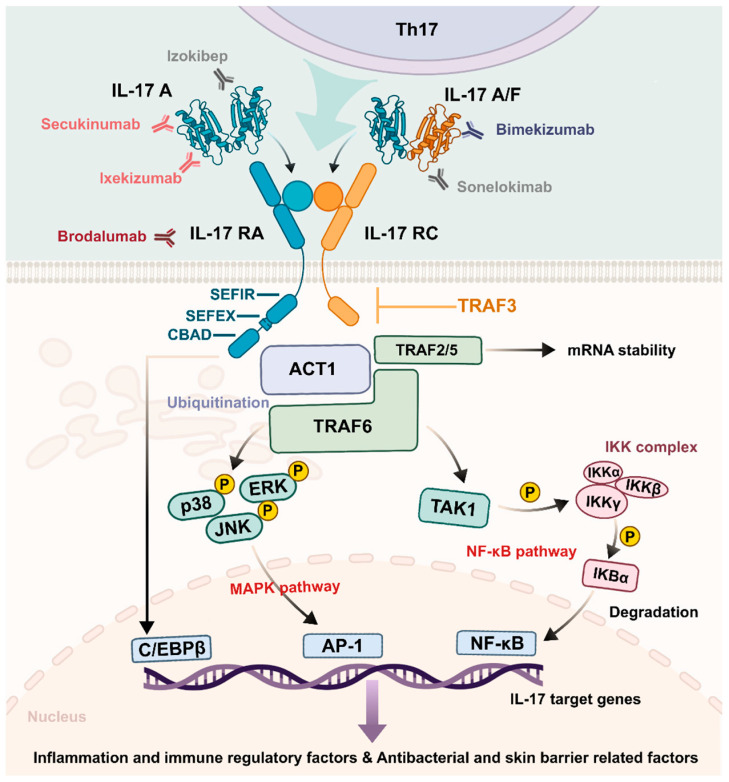
IL-17A/F signaling pathway and targeted therapeutics. IL-17 binds to the IL-17RA/RC receptor complex, which exists as a heterodimer on target cells. Upon ligand binding, the adaptor protein Act1 is recruited to the receptor via SEFIR domain interactions, leading to the formation of an Act1–TRAF6 complex. This complex activates downstream signaling cascades, including the NF-κB pathway via TAK1–IKK complex formation and the MAPK pathways via ERK, p38, and JNK. Meanwhile, IL-17 signaling can directly activate C/EBPβ via the CBAD, further enhancing transcriptional responses. The signaling pathways activate gene transcription, which produces pro-inflammatory mediators and antimicrobial peptides. TRAF2/5 protects mRNA from degradation, while TRAF3 serves as a critical negative regulator of IL-17 signaling. Secukinumab, Ixekizumab, Brodalumab, and Bimekizumab are biologic agents targeting IL-17 isoforms and IL-17 receptor subunits for treatment purposes; Sonelokimab and Izokibep represent experimental drugs not received approval for medical applications. MAPK: mitogen-activated protein kinase; NF-κB: nuclear factor-κB; C/EBPβ: CCAAT/enhancer-binding protein β; CBAD: C/EBPβ activation domain.

The IL-17A and IL-17F signaling pathway uses a heterodimeric receptor complex, which contains IL-17RA and IL-17RC subunits to transmit signals [22]. The IL-17 receptor family has a SEF/IL-17R (SEFIR) domain serving as its characteristic intracellular structure [23] (Figure 1). This domain lacks the critical residues required for MyD88 recruitment, thereby mechanistically distinguishing IL-17 signaling from the canonical pathways utilized by most Toll-like receptors and other adaptive immune receptors.

The IL-17RA/RC receptor complex requires Act1 [24] as its necessary cytosolic adaptor protein to execute its function following ligand attachment. The E3 ubiquitin ligase function of Act1 brings TRAF6 into a ubiquitination reaction that results in K63-linked ubiquitination. The Act1–TRAF6 pathway activation causes standard inflammatory responses through NF-κB activation via TAK1–IKK complex formation and MAPK pathway activation via ERK, p38 and JNK [25]. The signaling process leads to increased production of essential pro-inflammatory cytokines (e.g., IL-6 and TNF-α), chemokines (e.g., CXCL1 and CXCL8), and antimicrobial peptides (e.g., β-defensins), being required for the first line of defense. IL-17 signaling activates CCAAT/enhancer-binding protein β (C/EBPβ) through its non-conserved C/EBPβ activation domain (CBAD) [26,27], which is found at the distant intracellular section of IL-17RA to regulate specific inflammatory genes and metabolic pathways.

Rather than depending solely on TRAF6 to drive transcription, IL-17 signaling draws on several other proteins within the same TRAF family to ensure the message is fully delivered. Act1 recruits TRAF2 and TRAF5 to form a regulatory complex that binds to the 3′ untranslated regions (3′ UTRs) [28] of mRNAs encoding chemokines and MMPs, such as CXCL1 [29]. The unstable transcripts maintain their stability through interactions with their binding partners, which protects them from Regnase-1 [30] endoribonuclease degradation that leads to stable mRNA expression and prolonged inflammatory mediator production in wound tissue. Meanwhile, TRAF3 serves as a critical negative regulator of IL-17 signaling [31,32]. By binding to the distal intracellular domain of IL-17RA, TRAF3 disrupts the Act1–TRAF6 interaction, establishing a negative feedback loop that restrains excessive IL-17–driven inflammation and limits collateral tissue damage [33]. The signaling modules function as a single system to generate IL-17, which serves as an immunoregulatory complex that connects immediate inflammatory responses to permanent tissue repair processes.

### 2.2. Type 17 Immune Cells

The production of IL-17 during cutaneous wound healing and inflammatory skin disorders happens through different immune cells. Based on developmental origin and functional specialization, IL-17–producing cells can be broadly classified into three categories: adaptive lymphocytes, innate-like lymphocytes, and myeloid cells, which are called “Type 17” immune cells [11].

The innate-like lymphocytes are ancient Type 17 immune system components, acting as the first line of defense. Although adaptive lymphocytes are not among the earliest responders during the initial hours following acute injury, they constitute the principal source of IL-17 during the later phases of inflammation and in chronic inflammatory conditions. The dermal tissue contains CD4^+^Th17 cells, which represent the typical producers of IL-17. The cutaneous Th17 cells, as an immune hub connects defensive responses against extracellular pathogens to neural signals and metabolic signals in the skin. IL-17–producing CD8^+^ T cells, called Tc17 cells, have been identified as prominent contributors in psoriatic lesions and certain forms of contact dermatitis [34]. The epidermal tissue contains tissue-resident memory T cells (TRMs) located at the skin surface and dermo–epidermal junction to enhance local Type 17 immune responses through IL-17 production after antigen detection, which leads to keratinocyte growth and antimicrobial peptide production [35].

A large number of γδ T cells [36] are found in the skin of mice, which start producing IL-17A through stress signal detection and inflammatory cytokine response within short time periods before Th17 cells are activated [37]. However, the proportion of γδ T cells in human skin is much lower than that in mice. Dendritic epidermal T Cells, a specific subset of epidermal γδ T cells unique to mice, in mice regulate skin antimicrobial barrier function via IL-17A, inducing keratinocytes to produce antimicrobial peptides [38]. Group 3 innate lymphoid cells (ILC3s) [39] lack antigen-specific receptors, are strategically positioned at barrier surfaces and integrate cytokine cues such as IL-23 and IL-1β to secrete IL-17 and IL-22 to improve epithelial integrity and repair [40], supported mainly by murine and limited human data. In human skin, mucosal-associated invariant T (MAIT) cells constitute another important source of IL-17 [41]. These cells respond to microbially derived metabolites and have been implicated as early responders in infectious settings and metabolically compromised wounds.

In addition, innate myeloid cells can serve as substantial sources of IL-17, particularly under pathological conditions. In psoriasis, neutrophils and mast cells have been identified as major IL-17 reservoirs. Neutrophils can rapidly release preformed IL-17 through the neutrophil extracellular traps (NETs) [42,43]. NETosis helps to contain microorganisms but the process of NET formation becomes a problem when it occurs excessively or uncontrollably, causing an intense inflammatory response that hinders wound healing. Similarly, mast cells contain IL-17 in their cytoplasmic granules, releasing it through degranulation to create an instant pro-inflammatory cytokine response during tissue damage [44]. Studies also show that newly identified Ly6c(lo)MHCII(hi) macrophages in murine wound models increase in both proportion and absolute number during wound healing, producing low expression of IL-17A [45].

The immune response of IL-17 in wound healing functions as a time-dependent signaling system, which starts with tissue-resident immune cells, including γδ T cells, MAIT cells and mast cells, followed by Th17 and Tc17 cell recruitment for extended IL-17 production. Spatially and temporally regulated Type 17 immune cells enable fast antimicrobial defense and extended immune monitoring, which also affect tissue repair and remodeling processes. It must be acknowledged that this conclusion is well-supported by evidence in the mouse trauma model, but lacks longitudinal dynamic tracking data in human acute trauma cases, because human data mostly come from chronic wounds or psoriasis.

## 3. Protective Mechanisms in Acute Wound Repair

### 3.1. Early Inflammation and Pathogen Clearance

**Figure 2:**
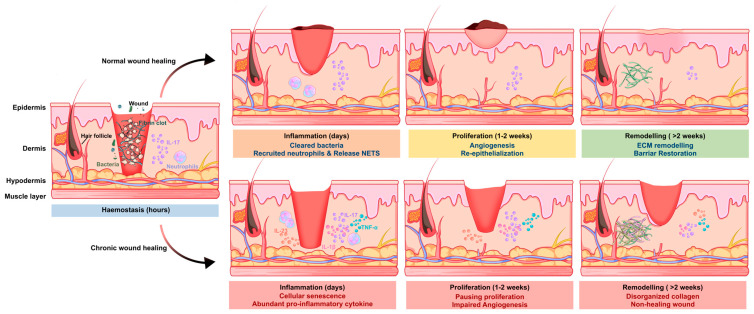
The dual role of IL-17 in acute versus chronic wounds. The upper panel shows the protective pathway during typical acute tissue healing processes. In the early inflammatory phase, IL-17 promotes host defense by inducing chemokine production, recruiting neutrophils to the wound site to eliminate invading pathogens. During the subsequent proliferative phase, IL-17 enhances keratinocyte proliferation and migration to facilitate re-epithelialization, contributes to angiogenesis through the induction of pro-angiogenic mediators, and supports fibroblast activation and ECM deposition. These coordinated effects promote efficient tissue repair and remodeling. The lower panel depicts the pathological consequences of sustained IL-17 activation in chronic wounds. Chronic IL-17 activity amplifies neutrophil infiltration, increases the production of pro-inflammatory cytokines, and drives continuous release of proteolytic enzymes, resulting in excessive ECM degradation. Persistent inflammatory stress further induces cellular senescence in keratinocytes and fibroblasts, impairs angiogenesis, and disrupts tissue remodeling. These mechanisms prevent proper wound closure and contribute to the transition from acute to chronic, non-healing wounds. AMPs: antimicrobial peptides; ECM: extracellular matrix.

During the early phase of wound healing, IL-17 functions not only as a pro-inflammatory cytokine but also as a key coordinator that links immune system defense to the beginning of tissue restoration [13]. By promoting rapid pathogen clearance and amplifying localized inflammatory signaling, IL-17 helps establish a controlled, microbe-restricted wound environment that is essential for subsequent regenerative processes (Figure 2).

The first necessity for early wound healing involves creating an environment against microbial invasion. IL-17 is one of the most potent inducers of neutrophil mobilization. IL-17 causes resident fibroblasts, endothelial cells [46], and keratinocytes [47,48] at the wound border to produce CXC chemokines [49], thereby facilitating the rapid recruitment of circulating neutrophils to sites of tissue injury. The host defense system benefits from recruited neutrophils [50] because they perform phagocytosis and they produce NETs [51] through IL-17 signaling, which effectively captures and eliminates typical wound bacteria. IL-17 activates epithelial cells to generate different antimicrobial peptides (AMPs) through its direct stimulation of these cells. Keratinocytes are also activated to produce β-defensins (hBD-2 and hBD-3) [52] and release S100 family proteins and lipocalin-2 (Lcn2), as nutritional immunity factors to block essential metal ions from microbes, stopping their growth, bacterial attachment, and biofilm formation. In addition, keratinocyte-derived IL-17C reinforces this antimicrobial barrier through autocrine and paracrine signaling to establish a local positive feedback loop that sustains early innate defense [53,54].

IL-17 enhances inflammatory reactions through its strong interaction with danger-associated signals, which tissue damage releases (e.g., TNF-α and IL-1β) and microbial compounds (e.g., LPS), which produce fast cytokine and chemokine accumulation during the initial hours of injury [55]. The body needs this strong inflammatory response to heal wounds properly during acute wound repair but it becomes harmful in chronic conditions. It enables efficient clearance of necrotic debris and invading microorganisms, and secures the timely transition from the inflammatory phase to the proliferative phase of healing.

### 3.2. Re-Epithelialization and Tissue Regeneration

IL-17 signaling as a vital factor during the proliferative phase promotes wound healing through its role in tissue regeneration and re-epithelialization. The downstream effector regenerating islet-derived protein 3 alpha (REG3A) functions as a C-type lectin, which serves two main purposes by defending against microbes and strongly stimulating keratinocyte cell division [56,57]. The IL-17A receptor complex activates the Act1–TRAF6 signaling pathway, causing SHP-2/ERK pathway activation that produces high levels of REG3A gene expression. The REG3A protein could prevent keratinocyte maturation while it keeps cells in an abnormal state of continuous cell proliferation. The brief hyperproliferative state, which looks like psoriasis, allows the wound to get quick coverage through its initial epithelial healing mechanisms.

Research on metabolic processes shows that IL-17 signaling causes major changes in the metabolic functions of wounded epithelial tissue. IL-17 triggers the AKT–mechanistic target of rapamycin (mTOR) pathway to stabilize HIF-1α protein through this process [58]. The signaling pathway leads keratinocytes at the wound border to switch their metabolism toward glycolysis, which generates the necessary energy for their fast movement and tissue repair.

In addition to driving proliferation and metabolism, IL-17 contributes to the restoration of epithelial barrier integrity. An IL-17C–dependent autocrine loop in keratinocytes induces the expression of tight junction proteins, including occludin, claudin-1, claudin-4, and zonula occludens-1 (ZO-1). The junctional complexes function as “zippers”, enhancing intercellular adhesion to prevent microbial invasion and reduce excessive fluid loss from interstitial spaces. As a barrier-restorative mechanism, the system preserves local microenvironmental stability during the last phase of epithelial tissue healing.

Furthermore, Tc17 cells elicited by skin commensal microorganisms secrete amphiregulin (AREG), a ligand for the epidermal growth factor receptor (EGFR) [59]. The AREG–EGFR interaction activates two complementary signaling pathways [60,61]. The RAS–RAF–MAPK/ERK cascade activation causes cells to advance through their cell cycle from G1 to S phase, which produces elevated numbers of epithelial cells. While the PI3K–AKT–mTOR pathway activation works together with IL-17-induced metabolic processes to increase protein production and glycolytic activity. Together, these coordinated signaling networks provide the molecular and metabolic foundation for efficient epithelial regeneration.

### 3.3. Angiogenesis and Sensory Nerve Repair

The process of restoring tissue integrity needs not only epithelial wound closure, but also the recovery of blood vessel function and nerve supply, which IL-17 family cytokines strongly affect. IL-17 works together with TNF-α to create a synergistic effect in increased production of VEGF, GM-CSF and CXCL8 by fibroblasts and keratinocytes during angiogenesis [62]. The mediators work together to stimulate endothelial cell growth, movement, and the formation of tubular structures. Moreover, IL-17–mediated stabilization of HIF-1α further amplifies angiogenic signaling, ensuring adequate oxygen and nutrient delivery to the metabolically demanding regenerating tissue [63,64].

The role of IL-17 signaling in sensory nerve regeneration is equally critical, which is often underappreciated [65,66]. Research landmark studies have proven that Th17 and Tc17 cells induced by skin commensals secrete IL-17A, enhancing both tissue repair and nerve fiber restoration after damage, with the cooperation of amphiregulin in mice [67,68]. The injured keratinocytes release IL-17C, which may protect neurons from death while their axons grow into new connections to establish an independent epithelial–neural regenerative pathway [69].

IL-17A produces two main effects on the nervous system through its direct action on neurons. IL-17RA is widely expressed on nociceptive sensory neurons within the dorsal root ganglia [70]. The activation threshold of peripheral nerve terminals becomes lower at tissue injury sites because IL-17A signaling leads to increased mechanical sensitivity. The IL-17-mediated hyperalgesia in acute wounds is a protective system that protects tissues from additional harm [71]. The body develops negative effects from IL-17 signaling when inflammation becomes persistent because this results in abnormal nerve sensitivity, which leads to chronic neuropathic pain development [72,73].

## 4. Pathogenic Roles in Chronic Pathological Conditions

### 4.1. Sustained Inflammatory Amplification and Cellular Senescence

IL-17 is an initial repair signal in chronic wounds but it later becomes the main factor that maintains ongoing inflammation in diabetic foot ulcers. The disease state exists because Th17 cells stay active for extended periods, while innate cells producing IL-17, such as neutrophils and mast cells, take a long time to disappear [13]. The extended inflammatory response results from the stabilization of pro-inflammatory mRNA transcripts, and continues to produce chemokines and cytokines at high levels. The current environment maintains a self-perpetuating loop between IL-23 and IL-1β, sustaining the disease-inducing Th17 immune response while preventing the formation of protective regulatory cells.

The body produces reactive oxygen species (ROS) in keratinocytes and fibroblasts located at the wound margin when IL-17 levels remain high throughout time [74,75]. The resulting oxidative stress activates the DNA damage response (DDR), causing early cellular senescence through p53/p21 pathway activation [76,77]. The cells that stop growing but continue metabolic activity develop the senescence-associated secretory phenotype (SASP), including the production of pro-inflammatory cytokines (e.g., IL-6 and IL-8) and matrix-degrading enzymes [78]. SASP factors block epithelial cell movement while creating an unfriendly environment, which makes the wound more resistant to treatment [79,80,81].

### 4.2. Tissue Damage and Impaired Barrier Function

The process of protein breakdown becomes abnormal in the development of chronic wounds. The continuous IL-17A signaling in diabetic ulcers leads to excessive collagenase and gelatinase production from immune cells, including macrophages, neutrophils, and stromal cells, but it does not trigger sufficient production of their built-in inhibitors known as tissue inhibitors of metalloproteinases (TIMPs) [82]. The unbalanced state between catabolic and anabolic processes leads to faster ECM breakdown, creating a condition that blocks wound healing through its inability to achieve closure. The process of excessive proteolysis creates an environment that hinders angiogenesis from occurring. The degradation of VEGF and other growth factors, including PDGF and TGF-β, prevents their biological functions, and IL-17, together with TNF-α, causes endothelial cells to experience oxidative stress, leading to their death and causing the destruction of new capillary networks [83].

IL-17 signaling maintains its activity throughout time, which results in damage to the skin barrier function. IL-17 causes the decrease of filaggrin and structural proteins, leading to parakeratosis that resembles psoriatic plaque formation and produces fragile neo-epidermis [84,85]. Administration of recombinant IL-17A in acute wounds has been reported to delay healing by inducing excessive neutrophil elastase activity [86]. Persisting neutrophils will undergo NETosis to produce elevated levels of IL-17 and cytotoxic histones that further exacerbate tissue injury.

### 4.3. Tissue Fibrosis and Scar Formation

In the late remodeling phase, a high level of IL-17 culminates in pathological fibrosis. IL-17 drives fibroblast cells to develop into myofibroblasts, which perform the main functions of collagen accumulation and wound tightening through direct or indirect mechanisms that include IL-6 as a mediator [87]. Concurrently, IL-17 synergizes with TGF-β to amplify Smad2/3 phosphorylation and induce profibrotic gene expression, disrupting ECM architecture [88].

The IL-17 signaling pathway prevents adipose tissue regeneration because it stops preadipocyte development through decreased expression of PPARγ and C/EBPα genes [89]. IL-17 activates TLR4, inducing lipolysis and atrophy of fully developed adipocytes. The process of tissue regeneration produces two main problems, which result in decreased adipose tissue formation and excessive fibroblast cell growth that leads to scar tissue development. This imbalance shifts the wound healing trajectory from regenerative repair toward pathological fibrosis and functional impairment.

## 5. Th17 Plasticity and Stromal Regulation

### 5.1. Th17 Cell Plasticity and Transcriptional Regulation

**Figure 3:**
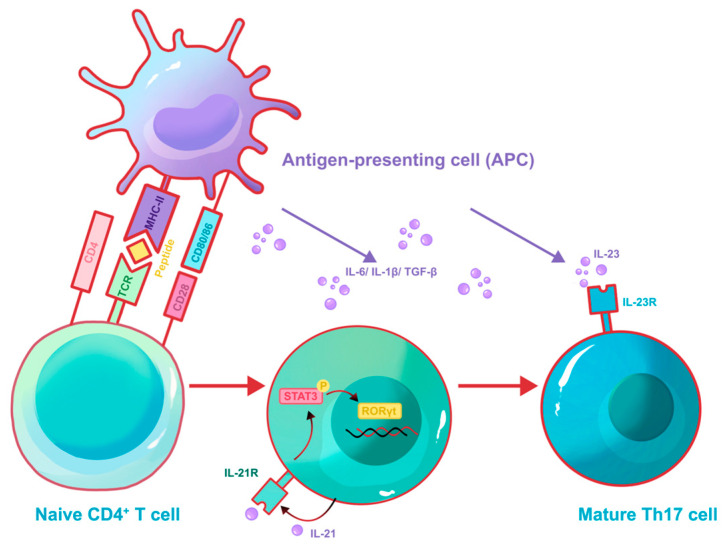
Differentiation of Th17 cells. During the priming phase, upon recognition of antigen presented by an antigen-presenting cell (APC) via MHC-II and costimulatory signals (e.g., CD80/86 binding to CD28), naïve CD4^+^ T cells are driven by the combined action of transforming growth factor-β (TGF-β) and IL-6 (and IL-1β, as shown in the figure) to initiate intracellular signaling cascades (e.g., MAPK and STAT3 activation). This signaling promotes the expression of the transcription factor RORγt and autocrine cytokine IL-21, which further amplifies STAT3 signaling via the IL-21 receptor. The sustained activation of STAT3 and RORγt reinforces the Th17 differentiation program, leading to upregulation of the IL-23 receptor (IL-23R) and enabling full maturation in response to IL-23. Ultimately, the committed Th17 cells produce signature cytokines such as IL-17 and IL-22.

It was previously assumed that Th17 cells possessed a fixed identity that didn’t switch or adapt once formed [90]. During the priming phase, upon recognition of antigen presented by an antigen-presenting cell (APC) via MHC-II and costimulatory signals, naïve CD4^+^ T cells are driven by the combined action of transforming growth factor-β (TGF-β) and IL-6 to induce expression of the lineage-determining transcription factor retinoic acid receptor–related orphan receptor-γt (RORγt), thereby committing to the Th17 differentiation program and producing signature cytokines such as IL-17 and IL-22 (Figure 3). The last ten years have brought major changes to this understanding because scientists now show that Th17 cells exist as a flexible immune type instead of being a terminally differentiated type, whose plasticity is governed by the local cytokine milieu [91]. In the progress of wound healing and skin repair, Th17 cells typically polarize into two distinct functional states: a pathogenic “Th1-like” phenotype that fuels chronic inflammation, or a regulatory “Treg-like” phenotype that facilitates tissue resolution and remodeling (Table 2).

In human chronic cutaneous inflammation, Th17 cells can progressively downregulate RORγt while upregulating the Th1-associated transcription factor T-bet, thereby acquiring the capacity to co-produce IL-17 and interferon-γ (IFN-γ) [92,93]. The population known as Th1-like Th17 cells shows RORγt^+^T-bet^+^ double-positive transcriptional expression and it remains less sensitive to Treg cell-mediated suppression [94]. Persistent Th1-like Th17 cells prevent the body from building proper granulation tissue and repairing the epithelial layer and extracellular matrix. IL-23 functions as the main factor that drives this disease progression by sustaining RORγt expression through STAT3 signaling pathways and blocking Foxp3 re-expression [95,96]. IL-12 enables cells to acquire IFN-γ, while IL-1β in the inflammatory environment both strengthens and speeds up this cell type transformation [97].

**Figure 4:**
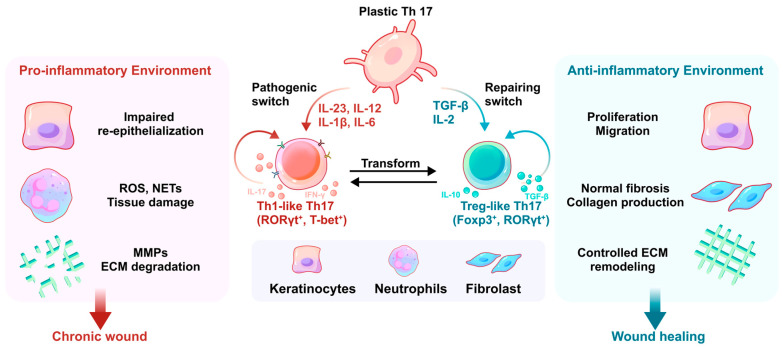
Th17 cell plasticity during wound healing. Th17 cells show functional plasticity due to specific cytokines and metabolic signals that exist in the wound area. Elevated levels of IL-23, IL-12, IL-1β, and IL-6 promote the transition of Th17 cells toward a Th1-like phenotype, which is characterized by increased expression of T-bet and enhanced production of IL-17 and IFN-γ. This phenotypic shift amplifies pro-inflammatory responses, thereby contributing to delayed healing or chronic wound formation. Conversely, during the mid-to-late stages of physiological wound repair, a microenvironment enriched in TGF-β and relatively depleted of pro-inflammatory cytokines causes Th17 cells transdifferentiate toward a Treg-like phenotype, characterized by increased Foxp3 expression, which is critical for restoring tissue homeostasis and facilitating resolution of inflammation.

The process of wound healing needs Th17 cells to shift toward a regulatory phenotype for successful inflammation resolution. The absence of IL-6 and IL-23 in TGF-β-enriched microenvironments allows Th17 cells to transform into Treg-like Th17 cells [98]. The process leads to Foxp3 gene activation and IL-10 anti-inflammatory cytokine production increases. The Treg-like Th17 cells execute various wound healing tasks because they manage uncontrolled inflammation and assist keratinocytes in their development and skin tissue reconstruction and stimulate fibroblast activity for extracellular matrix remodeling [99]. The phenotypic transformation of cells also depends on their surrounding metabolic environment, which will be explained in detail later (Figure 4).

### 5.2. Atypical Cellular Regulation of IL-17 Signaling

The persistence of IL-17–driven inflammation is also sustained through reciprocal positive feedback loops between immune cells and tissue-resident stromal cells. Keratinocytes function not merely as passive targets of IL-17 but as active amplifiers of IL-17 signaling. IL-17A activation of keratinocytes results in the immediate production of CCL20 (macrophage inflammatory protein-3α, MIP-3α) chemokine through NF-κB and Syk–CARMA2 signaling pathway [100]. The chemokine CCL20 functions as the only binding molecule for CCR6, which exists in high amounts on Th17 and Tc17 cells, thus creating a strong chemokine signal that draws Type 17 lymphocytes into the epidermal tissue. The IL-17–CCL20–CCR6 feed-forward circuit operates as a fundamental mechanism that determines the development of chronic inflammatory skin diseases that affect psoriasis and non-healing cutaneous wounds. Fibroblasts develop into pro-inflammatory cells producing IL-6, IL-8 and GM-CSF when they receive prolonged IL-17 exposure. These mediators enhance the survival, proliferation, and activation of neutrophils and Th17 cells in turn, thereby reinforcing the inflammatory milieu. Mechanistically, IL-17 signaling in fibroblasts works with TNF-α to maintain mRNA stability, producing these inflammatory factors [101]. The post-transcriptional regulation depends on RNA-binding proteins Act1 and HuR, which protect their target transcripts from degradation while prolonging the production of cytokines [102].

The pathogenic network receives additional support from IL-17C epithelial cells produced [53]. IL-17C emerges from keratinocytes, which respond to tissue damage and inflammatory signals, then stimulates IL-17A production in epithelial cells and leukocytes that enter the tissue [54,103], supported mainly by murine models and in vitro human keratinocyte studies. The resulting cytokine circuitry may create a complex system of self-sustaining inflammation, which keeps wounded tissue stuck in continuous inflammation. The disruption of stromal–immune crosstalk suggests potential as an effective treatment approach helping eliminate IL-17-dependent chronicity while enabling proper wound healing processes, which are largely extrapolated from preclinical models.

## 6. Microenvironmental Regulatory Networks of IL-17 Signaling

**Figure 5:**
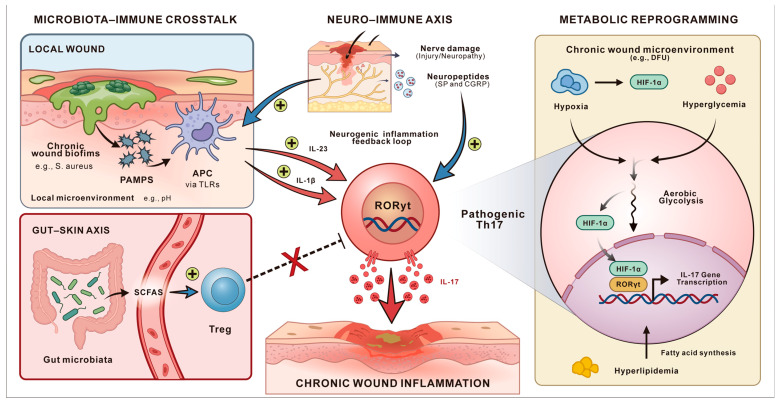
The microenvironment regulates IL-17 signaling pathways through various control systems that operate at different levels. The diagram shows how non-healing chronic wounds develop through three connected systems, which include (**Left**) The Microbiota−Immune Axis: Local biofilms and the gut microbiota work together to control the Th17 phenotype. (**Center**) The Neuro−Immune Axis: Neuronal injury and aberrant signaling pathways establish a neurogenic inflammation feedback loop. (**Right**) Metabolic Reprogramming: The combination of hypoxia, hyperglycemia and hyperlipidemia creates conditions that lead to the development of pathogenic Th17 cells and maintain ongoing inflammation.

IL-17 signaling is not isolated but is intricately shaped by a network of local and systemic microenvironmental cues. In chronic wounds, microbial community, neural signals, and metabolic status collectively regulate the balance between pathogenic and reparative Type 17 responses, determining whether inflammation persists or tissue repair proceeds (Figure 5).

### 6.1. Microbiota-Immune Crosstalk

The local microbial community of chronic wounds shows two main characteristics, which include dysbiosis and polymicrobial biofilms, frequently dominated by pathogens [104]. Increased abundance of *Staphylococcus aureus* has been associated with delayed re-epithelialization and heightened inflammatory cytokine expression, whereas *Pseudomonas aeruginosa* biofilms correlate with persistent neutrophilic inflammation and impaired granulation tissue formation [105]. In addition, reduced microbial diversity and dominance of anaerobic genera such as *Finegoldia* and *Anaerococcus* have been linked to poor healing trajectories in diabetic foot ulcers [106,107]. The biofilms maintain persistent PAMPs, including peptidoglycan and LPS, that activate TLRs on antigen-presenting cells (APCs) throughout the entire process, as suggested in experimental models. This chronic stimulation drives constitutive secretion of IL-23 and IL-1β, which supports the pathogenic Th1-like Th17 cells while blocking the development of Treg-like cells needed for tissue repair [108].

However, recent research shows the microbial effects are highly context-dependent in wound healing progress, depending on local microenvironment, such as oxygenation, pH, and the host’s immune tone [109]. In the alkaline environment of chronic wounds (pH > 7.5), *Pseudomonas aeruginosa* significantly upregulates its quorum-sensing pathways, leading to increased production of virulence factors like pyocyanin and elastase [110]. These factors degrade host growth factors and the ECM, effectively stalling the repair process. In diabetic murine wound models [111], hyperglycemic conditions are associated with increased bacterial burden, denser biofilm formation, prolonged inflammation, and delayed wound healing, indicating that hyperglycemia may impair wound healing by weakening host immunity and indirectly favoring bacterial persistence [112].

Systemic microbiota also influences cutaneous immunity via the gut–skin axis, which is often indirect. The intestinal bacteria, such as segmented filamentous bacteria (SFB) in mice, and other gut commensals trigger Th17 cell production in the intestine, and then move to distant locations, including the skin [113]. The fermentation of dietary fibers by microorganisms produces short-chain fatty acids (SCFAs), including butyrate that functions as a histone deacetylase (HDAC) inhibitor [114]. The body uses SCFAs to perform Foxp3 locus acetylation, which leads to Treg cell development and reduces Th17 cells that cause damage to tissues. Therefore, it could be simply speculated that systemic dysbiosis disrupts this metabolic balance, contributing to a pro-inflammatory systemic milieu that exacerbates local pathology [115]. However, the results have been proven only in studies using mouse models and in vitro PBMC cultures of humans.

### 6.2. The Neuro–Immune Axis

**Figure 6:**
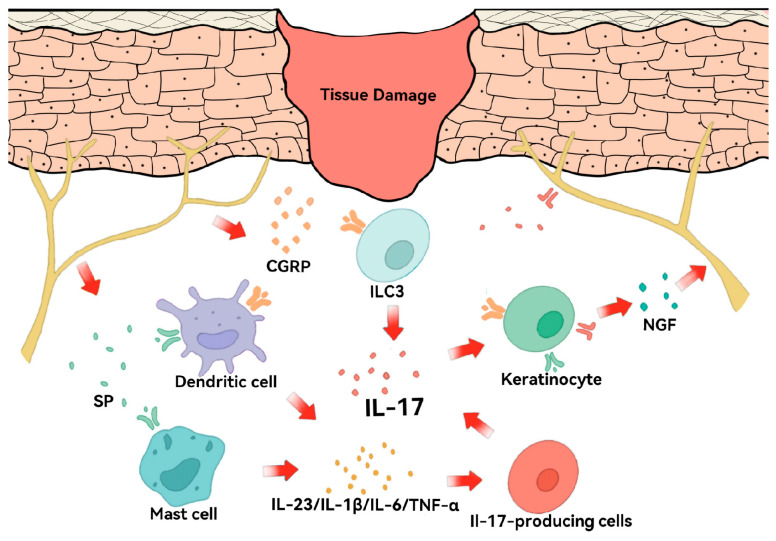
Schematic of the neuro–immune loop in skin repair. Tissue damage triggers the release of neuropeptides CGRP and SP from sensory nerve fibers. These mediators activate dendritic cells, mast cells, and ILC3, which subsequently produce key cytokines (IL-23, IL-1β, IL-6, TNF-α) and drive the activation of IL-17. Furthermore, IL-17 acts back on sensory neurons via IL-17 receptors and keratinocytes, modulating neuronal activity and neuropeptide release, thereby completing a critical feedback loop that integrates neural and immune responses for tissue homeostasis.

The peripheral nervous system plays an essential role in controlling immune responses in wound repair [116] (Figure 6). The body releases neuropeptides, including substance P (SP) and calcitonin gene-related peptide (CGRP), from nociceptors after tissue damage occurs [117]. The substance SP activates dendritic cells and mast cells while CGRP stimulates ILC3 to produce key cytokines (IL-23, IL-1β, IL-6, TNF-α) [118,119], which may maintain Th1-like Th17 cells in a state that drives the activation of IL-17 without requiring active infection. However, evidence regarding CGRP-mediated immune regulation is primarily derived from murine models. Therefore, this neuro-immune regulatory circuit should currently be considered a preclinical mechanistic model rather than a fully established pathway in human wound healing.

IL-17 can also interact with the nervous system through both direct and indirect mechanisms. In murine models of inflammatory pain and tissue injury, IL-17 receptors have been detected on sensory neurons, where IL-17 signaling can modulate neuronal excitability. In addition, IL-17 may indirectly influence neuronal activity and neuropeptide release through keratinocyte-derived neurotrophic factors such as nerve growth factor (NGF), thereby completing a critical feedback loop that integrates neural and immune responses for tissue homeostasis [120,121]. Although human keratinocytes can produce NGF in response to inflammatory cytokines in vitro, the complete IL-17–NGF–neuron feedback loop has not been fully validated in human skin tissue in vivo.

In diabetic neuropathy with DFUs, this axis is profoundly dysregulated: (a). Glucotoxicity causes peripheral nerve degeneration, which may lead to an insufficient IL-17 response during the initial stages, which compromises local antimicrobial defense and extending the duration of the inflammatory phase. (b). Residual damaged nerves, exposed to sustained IL-17, probably release pro-inflammatory mediators that perpetuate neurogenic inflammation, leading to the process of late sensitization and the hyper-inflammatory environment [122]. (c). Beyond the inhibitory effects of the chronic inflammation, tissue restoration may be further stalled by impaired IL-17C and Tc17 signaling, which blocks the growth of new nerve cells and prevents the tissue from obtaining essential immunomodulatory neuropeptides for its anti-inflammatory state. However, their roles in human diabetic wound neuro-regeneration remain largely speculative.

This establishes a self-perpetuating cycle: initial nerve damage impairs early immune defense and delays healing, while chronic IL-17 elevation simultaneously inhibits neural repair and drives pathological pain. Nevertheless, many components of this model are primarily supported by murine or in vitro experimental evidence, and their translational relevance to human chronic wound healing requires further investigation.

### 6.3. T Cell Metabolism and Th17 Functional Phenotypes

Th17 functional fate is tightly coupled to metabolic programming. Th17 cell fate is tightly coupled to metabolic programming. Most mechanistic insights into Th17 bioenergetics have been derived from murine models and in vitro T-cell differentiation systems. Effector Th17 cells generate their energy through glycolysis instead of oxidative phosphorylation (OXPHOS), which resting T cells and Tregs use for their energy needs [123]. The mTOR–HIF-1α axis has been identified as a central regulator of glycolytic reprogramming in Th17 cells, mainly in murine studies [124]. HIF-1α enhances the expression of glycolytic enzymes and promotes RORγt-dependent transcriptional programs while destabilizing Foxp3, thereby favoring Th17 differentiation over Treg development. While elements of this pathway have been observed in human T cells in vitro, direct in vivo confirmation in human skin or wound tissue remains limited.

Pathogenic Th17 cells have also been reported to depend on de novo fatty acid synthesis (FAS) for membrane biogenesis and effector function in murine systems. Acetyl-CoA carboxylase 1 (ACC1) is required for Th17 differentiation in mice, and pharmacologic ACC1 inhibition suppresses Th17 development in preclinical studies [125]. In contrast, Tregs preferentially utilize exogenous fatty acids for β-oxidation. Although lipid metabolites such as polyunsaturated fatty acids (PUFAs) and short-chain fatty acids (SCFAs) can modulate the Th17–Treg balance in experimental systems [126], evidence supporting this metabolic switch in human cutaneous wound environments remains sparse.

The diabetic wound environment supports glycolytic and pathogenic Th17 programming through three main factors, which include (1) Hypoxia: The insufficient blood flow creates conditions that allow HIF-1α to remain active, thus maintaining Th17 gene expression. (2) Hyperglycemia: The body starts producing more glucose through glycolysis because it receives excessive substrates, yet RORγt remains constant because of O-GlcNAcylation. (3) Hyperlipidemia causes lipid levels to rise, causing RORγt and ACC1 gene expression increase, resulting in Th17 cells developing into disease-causing cells, which described primarily in murine or in vitro studies.

IL-17 signaling also profoundly reconfigures the metabolic landscape of the wound stroma [127]. In keratinocytes, sustained high concentrations of IL-17 activate the mTOR/HIF-1α signaling axis [128], leading to a significant upregulation of the glucose transporter GLUT1 and key glycolytic enzymes. As a result, keratinocytes preferentially engage in glycolysis even under normoxic conditions. Meanwhile, excessive glycolytic flux generates large amounts of lactate, which accumulate in the local microenvironment and lower the pH [129]. The subsequent drop in pH disrupts intercellular tight junctions, leading to the skin barrier integrity. Furthermore, enhanced glycolysis competitively consumes the limited local glucose supply, placing underlying fibroblasts under relative glucose deprivation and thereby suppressing collagen synthesis.

Therapeutic strategies targeting metabolic dependencies have shown efficacy in preclinical autoimmune models, such as glycolysis inhibition, ACC1 blockade, or modulation of GLUT1 activity (Table 3). These methods only target the harmful Th17 cells while they either enhance reparative Tregs shows potential to bring back tissue equilibrium through specific targeting of pathogenic cells instead of using broad immunosuppressive treatments. Nonetheless, their selective targeting of pathogenic Th17 cells and therapeutic benefit in human cutaneous wound healing remain to be demonstrated in clinical studies.

## 7. Therapeutic Targets and Future Research Directions

### 7.1. The Double-Edged Sword and Translational Challenges of IL-17 Inhibition

The FDA has approved IL-17 ligand-targeting monoclonal antibodies (Secukinumab, Ixekizumab), IL-17 receptor-targeting monoclonal antibodies (Brodalumab) and the dual IL-17A/F inhibitor (Bimekizumab) [130,131] (Table 4). Those treatments show effective results in treating chronic autoimmune diseases like plaque psoriasis and ankylosing spondylitis, but their use in acute trauma and chronic wound care faces major challenges.

Systemic IL-17 blockade might be ineffective in wound healing. Excessive IL-17 drives immunopathology and tissue damage, but its acute and moderate presence is necessary for host defense, epithelial barrier integrity, and initiation of early repair. The research indicates that complete body IL-17 blockade would result in inadequate wound protection during the first phase of wound healing. The elimination of IL-17A genes and the antibody-based IL-17A blocking methods may lead to delayed wound recovery, because neutrophils reduce their movement and keratinocytes decrease their antimicrobial peptide production, which increases the risk of Staphylococcus aureus and Candida albicans infections in animals [132]. The clinical data confirms these findings because patients who get IL-17 inhibitors develop mucocutaneous candidiasis more frequently, proving the essential role of IL-17 in human antifungal defense mechanisms. In complex conditions such as diabetic foot ulcers (DFUs), systemic blockade may disrupt metabolic homeostasis, further complicating disease management. Therefore, the process of maintaining IL-17 homeostasis requires exact control.

However, it must be acknowledged that therapeutic strategies capable of achieving precise spatial and temporal modulation of IL-17 still face substantial translational barriers.

- Lack of reliable real-time biomarkers. At present, there are no standardized markers that can clearly and dynamically distinguish between “beneficial” IL-17 signals (which support antimicrobial defense and early repair) and “harmful” IL-17 signals (which sustain chronic inflammation). Without such an indicator, such as the IL-17/IL-10 ratio, it is difficult to determine the optimal timing, dosage, and personalized decision-making for different patients [133].
- Challenges in delivery technology. Although responsive hydrogels and nanoparticle systems have been explored, achieving stable and controlled local drug release within the complex and changing wound environment, avoiding systemic exposure, presents significant technical challenges [134]. Proteases, bacterial biofilms, and fluid turnover in the wound bed may degrade or block the small therapeutic agents before they reach their target cells [135].
- Complexity of the IL-17 signaling network. IL-17 signaling is profoundly influenced and synergized by upstream factors and other environmental signals, interacting with various pathways [29]. Simply blocking one component may not produce the expected effect and could disrupt other protective mechanisms. Therefore, modulating IL-17 must consider the overall balance of the immune microenvironment, not just the endpoint of wound closure.
- Etiological and patient heterogeneity. Different wound types, such as diabetic foot ulcers and pressure ulcers, arise from distinct pathological processes, which means a single regulatory strategy is unlikely to be universally effective for all chronic wounds. In addition, patients—especially those with metabolic disorders like diabetes—show significant variation in immune status and wound microenvironment [136,137]. This high degree of heterogeneity makes it extremely difficult to establish standardized treatment protocols to achieve personalized treatments, including universal therapeutic windows or inhibition thresholds.

### 7.2. Wound-Specific Strategies Toward Precision Immunomodulation

The development of future strategies to manage systemic immunosuppression limitations needs precise delivery of immunomodulators to wound areas during active pathogen presence. Although clinical translation remains far from clinical application, current research has already begun to outline possible directions and future opportunities for development. However, it is important to note that most of these strategies have not been specifically validated in the context of IL-17 modulation in human wounds.

- Advanced Biomaterial Platforms. As delivery systems, bioengineered scaffolds use hydrogels and nanomaterials to provide controlled release of therapeutic agents [138]. Some studies have proposed that graphene oxide (GO)-based dressings incorporating microRNAs or small molecule inhibitors might influence inflammatory signaling pathways and angiogenesis at wound sites in order to promote new blood vessel growth. Nevertheless, direct evidence demonstrating that these materials can reliably and specifically regulate IL-17 signaling in vivo remains limited. Similarly, stimuli-responsive systems designed to release therapeutic agents in response to elevated inflammatory markers, such as matrix metalloproteinases (MMPs), represent a promising concept [139].
- Exosome-Mediated Modulation. Exosomes derived from mesenchymal stem cells (MSCs) contain miR-192-5p, which stems from adipose-derived stem cells (ADSC-Exo) to reduce fibrosis while promoting tissue healing through its direct inhibition of IL17RA expression in mouse fibroblasts [140]. This treatment method blocks Smad signaling and collagen accumulation in mice while it preserves the normal functioning of the immune system. However, their specific effects on IL-17–driven processes in chronic wounds remain incompletely understood.
- Metabolic Reprogramming. Research shows that pathogenic Th17 cells survive in hypoxic chronic wounds through HIF-1α regulation help them to perform aerobic glycolysis [128]. Based on this metabolic dependency, it has been hypothesized that localized glycolysis inhibitors or HIF-1α destabilizers might reduce persistent Th17 activity and metabolic shifts, and might promote the growth of anti-inflammatory regulatory T cells (Tregs). Furthermore, many biomaterials have been demonstrated in experimental models to promote wound healing by regulating immunometabolism through pathways involving mitochondrial function, ROS, and ferroptosis [141]. Still, such studies have only been validated under the specific metabolic conditions of wound healing, which cannot simulate the real and complex microenvironment.

It is necessary to integrate multi-modal data and computational approaches in the future. Single-cell RNA sequencing can analyze the heterogeneity of Type 17 immune cells, which exist as distinct populations. The technique of spatial transcriptomics enables scientists to locate these cells within particular tissue locations, which helps them understand how the surrounding tissue environment leads to disease development. Machine learning algorithms analyze local wound data together with systemic microbiome information and metabolomic results to create predictive models that doctors apply for individualized treatment planning to enhance patient results. The various treatment approaches show how precision immunomodulation can heal chronic wounds by using targeted methods that adjust IL-17 activity in particular wound environments.

## 8. Conclusions

The Th17/IL-17 axis, representing Type 17 immunity, produces two distinct effects that either help or harm the processes of wound healing and skin barrier restoration. IL-17 acts as a critical factor during the acute phase to achieve tissue regeneration through its role in pathogen elimination and its ability to stimulate epithelial tissue growth and blood vessel development and nerve sensation restoration. The IL-17 signaling pathway extends wound healing time in chronic or metabolically dysregulated conditions through its extended activation, leading to elevated inflammation and cellular aging and breakdown of the extracellular matrix and fibrosis formation.

The two distinct functions of Th17 cells exist because these cells demonstrate exceptional flexibility, which results from multiple control factors, including cytokines, metabolic signals, and microbiota presence and neural communication. The development of new therapeutic approaches needs to advance past the current approach involving either blocking or enhancing IL-17 activity. Future work should be placed on precise spatiotemporal immunomodulation strategies that remodel the local microenvironment to guide pathogenic Th17 cells toward reparative phenotypes, thereby providing a theoretical framework for immunotherapeutic strategies in refractory wound management.

## Figures and Tables

**Table 1:** Functional and structural distinctions of IL-17A, IL-17C, and IL-17F in the skin.

Feature	IL-17A	IL-17F	IL-17C
Gene	Chromosome 6p12.2	Chromosome 6p12.2	Chromosome 16q24.2
Molecular Structure	Homodimer (A/A) Heterodimer (A/F)	Homodimer (F/F) Heterodimer (A/F)	Homodimer (C/C)
Receptor Complex	IL-17RA + IL-17RC	IL-17RA + IL-17RC	IL-17RA + IL-17RE
Primary Cellular Sources	Th17 cells, Tc17 cells, γδ T cells, mast cells, ILC3s	Similar to IL-17A	Keratinocytes, epithelial cells, barrier-resident immune cells
Primary Dermal Targets	Keratinocytes, fibroblasts, endothelial cells, macrophages, neutrophils	Similar to IL-17A	Keratinocytes, epithelial cells
Intracellular Signaling	Activates MAPK, NF-κB, C/EBP signaling pathways via Act1/TRAF6 adaptors	Similar to IL-17A	Activates MAPK, NF-κB signaling pathways via Act1/TRAF6 adaptors
Major Functions	Central effector cytokine of Type 17 immunity	Similar but generally weaker induction of pro-inflammatory mediators compared to IL-17A	Epithelial homeostasis

**Table 2:** Key distinctions between pathogenic Th1-like Th17 cells and regulatory Treg-like Th17 cells in skin.

Feature	Pathogenic Th1-Like Th17 Cells	Regulatory Treg-Like Th17 Cells
Key Drivers	IL-6 + IL-1β + IL-23 + Low TGF-β1	TGF-β1 + IL-2
Transcription Factors	**Master:** RORγt **Co-expressed:** T-bet, STAT3, STAT4	**Master:** RORγt **Co-expressed:** Foxp3, c-Maf, AhR
Effector Cytokines	**High:** IL-17A, IFN-γ, GM-CSF **Co-expressed:** TNF-α, IL-22 **Low/absent:** IL-10	**High:** IL-10, IL-17A (lower levels) **Co-expressed:** TGF-β1 **Low/absent:** IFN-γ, GM-CSF
Surface Markers	CCR6^+^ CXCR3^+^, IL-23R	CCR6^+^ CXCR3^−^, CD25
Functional outcome	Tissue destruction, autoimmunity progression, neutrophil recruitment	Tissue repair, inflammation resolution, immune tolerance

**Table 3:** Metabolic iargets and iheir impact on the Th17/Treg axis in wound healing.

Target	Pathway	Intervention	Wound Outcome
mTOR/HIF-1a	Glycolysis	Inhibition: Promotes Treg	Resolution of Inflammation
ACC1/FAS	Fatty Acid Synthesis	Blockade: Impairs Th17	Reduced Tissue Damage
GLUT1/LDHA	Glucose Uptake Lactate Metabolism	Suppression: Reduces Lactate	Restored pH Balance

**Table 4:** Overview of IL-17-targeted therapeutic interventions.

Drug Name	Target and Mechanism	Indications	Clinical Status
Secukinumab	IL-17A fully human monoclonal antibody (IgG1κ)	Psoriasis (PsO), Psoriatic arthritis (PsA), hidradenitis suppurativa (HS)	Approved
Ixekizumab	IL-17A humanized monoclonal antibody (IgG4)	PsO, PsA, ankylosing spondylitis	Approved
Brodalumab	IL-17 receptor A (IL-17RA) receptor antagonist	Moderate-to-severe plaque psoriasis	Approved
Bimekizumab	IL-17A + IL-17F humanized dual-specific monoclonal antibody (IgG1)	PsO, PsA, HS	Approved
Netakimab	IL-17A humanized monoclonal antibody (IgG1)	PsA, moderate-to-severe plaque psoriasis	Approved (in Russia)
Sonelokimab	IL-17A + IL-17F trivalent nanobody (contains albumin binding domain)	PsO, HS	Phase II/III
Izokibep	IL-17A affibody molecule	HS, PsA	Phase III

## Data Availability

No new data were created or analyzed in this study.
